# Comparing the Effect of Dexmedetomidine Versus Intravenous Lidocaine on Colonoscopy Candidates Under Sedation with Propofol-Fentanyl: A Clinical Trial

**DOI:** 10.5812/aapm-138929

**Published:** 2023-12-23

**Authors:** Fatemeh Moftakhar, Reza Akhondzadeh, Fatemeh Hosseininejad, Sarina Alizade Ahvazi

**Affiliations:** 1 Department of Anesthesiology, Pain Research Center, Ahvaz Jundishapur University of Medical Sciences, Ahvaz, Iran; 2 Ahvaz Jundishapur University of Medical Sciences, Ahvaz, Iran

**Keywords:** Colonoscopy, Dexmedetomidine, Fentanyl, Lidocaine, Propofol, Sedation

## Abstract

**Background:**

Colonoscopy is an invasive and short-term diagnostic-therapeutic method that is associated with significant pain, discomfort, and anxiety in patients. Thus, various sedation and analgesia methods are used to reduce these complications.

**Objectives:**

This study compared the effect of dexmedetomidine versus intravenous lidocaine on colonoscopy candidates under sedation with propofol-fentanyl.

**Methods:**

This double-blind clinical trial was conducted on two groups of randomly divided patients (n = 60 each) referring to the colonoscopy unit of Imam Khomeini Hospital in Ahvaz, Iran. The first group was given 2% intravenous lidocaine with an initial dose of 1.5 mg/kg and a maintenance dose of 1 mg/kg/h, plus propofol 0.5 mg/kg and 1 µg/kg fentanyl. In contrast, the second group was given dexmedetomidine with an initial dose of 1 µg/kg and a maintenance dose of 0.5 µg/kg/h plus 0.5 mg/kg propofol and 1 µg/kg fentanyl. Hemodynamic changes, degree of sedation, and patients' pain were measured and recorded at certain intervals.

**Results:**

No significant differences were observed between the dexmedetomidine and lidocaine groups regarding gender, age, and weight (P > 0.05), and the two groups were homogeneous in this regard. The two groups were significantly different with respect to their heart rate after sedation (from 5 to 20 minutes) (P < 0.05), which was lower in the group receiving dexmedetomidine. In terms of mean arterial blood pressure, no significant difference was found between the dexmedetomidine and lidocaine groups (P > 0.05). With respect to the pain score at the end of the procedure, the two groups were significantly different (P < 0.05), with the group receiving dexmedetomidine obtaining a lower score.

**Conclusions:**

Although the use of lidocaine and dexmedetomidine is associated with the least hemodynamic changes, dexmedetomidine can create more suitable and favorable conditions during and after colonoscopy by inducing a higher degree of sedation and more analgesia.

## 1. Background

Colonoscopy is an endoscopic examination used to diagnose, screen, treat, and follow up on many colon diseases. Some patients can tolerate this procedure with no need for sedation, but for most patients, colonoscopy is an uncomfortable experience. Therefore, various techniques have been developed to relieve this pain and discomfort. Propofol-induced conscious sedation is the most commonly used method because of its optimal pharmacokinetic and pharmacodynamic properties, i.e., rapid onset, easy titration, and faster recovery (1). However, this type of sedation could be associated with respiratory depression, bradycardia, and dose-dependent hypotension. Propofol is a relatively new intravenous anesthetic that is very useful for inducing conscious sedation and has been associated with significant patient satisfaction. Propofol is a hypnotic drug-mediated by the gamma-aminobutyric acid (GABA) receptor and has a direct antiemetic effect. However, it does not have analgesic effects, and it seems that its combination with a short-acting narcotic is more effective. Fentanyl is a short-acting µ receptor agonist that can be used in combination with sleeping pills. It is a strong narcotic drug that is 75 - 125 times stronger than morphine and is used in different ways to induce analgesia and anesthesia (2).

FDA approved dexmedetomidine in late 1999 for human use as a short-term (less than 24 hours) drug to be used in the intensive care unit (ICU) for analgesia and sedation. Dexmedetomidine can be used in general and regional anesthesia as an anesthetic. It can also act as a prodrug, a sedative, and a pain reliever used after surgery (3). As a selective α-2 adrenergic receptor agonist (4), dexmedetomidine is characterized by anxiolytic, anesthetic, hypnotic, and analgesic properties (5). Through a negative feedback mechanism, this drug acts on presynaptic receptors and regulates norepinephrine release (4). The analgesic effects of dexmedetomidine have been attributed to alpha 2-adrenergic receptors, which inhibit the release of pain transmitters (i.e., substance P and glutamate) and spinal hyperpolarization (6). Dexmedetomidine has also been reported to decrease the rate of nausea, vomiting, and agitation (7). Overall, by inhibiting the release of norepinephrine, presynaptic activation of the adrenal α2 receptor terminates the transmission of pain signals. Sympathetic activity is inhibited by postsynaptically activated adrenal α2 receptors in the central nervous system (CNS), which can, therefore, reduce heart rate and blood pressure (8). Respiratory depression has not been associated with therapeutic doses of dexmedetomidine (9).

Its onset of action is less than 5 minutes, and the maximum effect occurs within 15 minutes (10). Lidocaine is an amide local anesthetic that not only inhibits G protein and Nmethyl-D aspartate (NMDA) receptors but also acts through sodium channel blockade (11). Intravenous administration of lidocaine leads to increased concentration of the neurotransmitter acetylcholine in the cerebrospinal fluid (CSF), and this can potentiate the nociceptive pathway (12) and possibly by binding to M3 muscarinic receptors (13), inhibiting glycine receptors (14), and secreting endogenous narcotics, it induces the final analgesic effect (15). Furthermore, as lidocaine comes into contact with the spinal cord, it will lead to direct or indirect reduction of postsynaptic depolarization through NMDA and neurokinin receptors (16). IV lidocaine has been found to be beneficial largely in visceral surgery because this drug relieves abdominal pain (17). Abdominal discomfort and visceral pain due to colonic distention during colonoscopy could be made tolerable by intravenous lidocaine (18, 19).

## 2. Objectives

The present study compared the effect of dexmedetomidine versus intravenous lidocaine on hemodynamic changes, degree of sedation, and the pain score of colonoscopy candidates under sedation with propofol-fentanyl.

## 3. Methods

This was a randomized clinical trial that obtained approval from the Medical Ethics Committee of AJUMS (IR.AJUMS.HGOLESTAN.REC.1401.156) and was registered on the Iranian Registry for Clinical Trials (IRCT20220706055402N2). This study was conducted on colonoscopy candidates in the colonoscopy unit of Imam Khomeini Hospital of Ahvaz, Iran, for 8 months in 2022. The study was a double-blind trial in which the data from questionnaires were recorded by a research assistant blinded to the drugs administered. Also, the attending physician of the present study was masked to group allocation. The participants were randomized into two groups of 60 according to their record number (random permutation of four).

The inclusion criteria included colonoscopy candidates aged 18 to 65 years who were class 1 or 2 based on the classification of the American Society of Anesthesiology (ASA). The exclusion criteria consisted of any history of addiction, severe heart or lung diseases, kidney or liver failure, allergy to study drugs, and pregnancy.

At the beginning of the patients' visit for the colonoscopy procedure, a written consent form was obtained, and the medical history of the patients was collected using the data in their medical records. After the patient's history was taken, the necessary examinations were performed, and the monitoring systems were connected. Then, the basic vital signs, including heart rate (HR), mean arterial blood pressure (MAP), and arterial blood oxygen saturation percentage (SPO_2_), were measured and recorded. A 20G intravenous catheter (B. Braun, Germany) was inserted, and oxygen was administered using a nasal cannula at 2 liters per minute.

The first group received intravenous lidocaine 2% (Caspian Tamin Pharmaceutical Co, Iran) with an initial dose of 1.5 mg/kg and a maintenance dose of 1 mg/kg/h plus propofol (Dongkook Pharm, Korea) 0.5 mg/kg and 1 µ/kg fentanyl (Caspian Tamin Pharmaceutical Co, Iran). The second group was given dexmedetomidine (Exir, Iran) with an initial dose of 1 µ/kg 10 minutes before the start of the procedure, followed by a maintenance dose of 0.5 µ/kg/h plus propofol 0.5 mg/kg and 1 µ/kg of fentanyl.

Throughout the procedure, MAP, HR, and SpO_2_ were measured and recorded every 5 minutes. Systolic blood pressure less than 90 mmHg or a drop of greater than 20% of the baseline value was regarded as hypotension. Appropriate serum therapy was used along with 5 mg of ephedrine to treat this complication.

Patients with bradycardia (HR < 50/min) received 0.5 mg of atropine. Respiratory depression was defined as SpO_2_ < 90%, which was treated with appropriate ventilation.

The degree of sedation of the patients was measured based on a modified Ramsay Sedation Scale before sedation and then every 5 minutes and recorded in the questionnaire. A Ramsay score of 5 or 6 was considered the optimal level of sedation, whereas a score below 5 represented an insufficient level of sedation. Additional propofol was administered if needed.

According to the modified Ramsay Sedation Scale, the patient:

(1) Is fully awake and anxious.

(2) Is sufficiently cooperative and tranquil.

(3) Sleeps and wakes up upon a verbal command.

(4) Sleeps and wakes up with mild stimulation but reacts strongly to painful stimulation.

(5) Has a sluggish reaction to painful stimuli.

(6) Does not react to painful stimuli.

Pain intensity was measured based on the VAS scale. A ruler graded from 0 to 10 (quantitatively discrete) was provided to the patients. According to this scale, 0 equaled no pain, scores between 1 and 3 represented mild pain, those between 4 and 6 indicated moderate pain, and scores between 7 and 10 showed severe pain. In the case of VAS scores above 3, 20 mg of intravenous meperidine (Aburaihan Pharmaceutical Co, Iran) was administered to control the pain. The degree of pain was measured and recorded at different intervals (at the start of the procedure, immediately after the procedure was finished, and one hour following the end of the procedure). Recovery time was considered from the time of completion of the procedure to the patients’ consciousness and appropriate answers to the questions. A period of less than 5 minutes was considered fast, between 5 - 10 minutes was considered medium, and more than 10 minutes was considered slow recovery.

Nausea and vomiting of patients were recorded from the end of the procedure to the time of leaving the recovery room. Considering that nausea is a complaint (symptom) and is expressed by the patients, the Visual Analogue Scale (VAS) method was used to measure and record it. A ruler graded from 0 to 10 (quantitatively discrete) was provided to the patients. 0: No nausea, 1 to 3: Mild nausea, 4 to 7: Moderate nausea, 8 to 10: Severe nausea. For ethical reasons, as soon as patients complained of moderate or severe nausea and vomiting, ondansetron was used with a therapeutic dose of 0.15 to 0.1 mg per kilogram of body weight and a maximum of 4 mg.

In this research, all participants were briefed on the study objectives and were assured that their information would remain confidential.

### 3.1. Sample Size Calculation and Sampling Method

Based on the objectives of the study, the opinion of the research team, and previous studies that examined the variable of pain (20), and assuming α = 0.05 and β = 0.9, d = 24, s = 15.5, a confidence level of 90%, and a power of 90%, we calculated the sample size as n = 60 for each group according to the following formula:


$$ni\;=\;\frac{2(z_{1-\frac{\alpha }{2}}+z_{\beta }\;)}{d^{2}}$$

where Z_1-α/2_ = 1.95, z_1-β_ = 1.95

### 3.2. Statistical Analysis

For quantitative variables, the mean was used to describe the central tendency of the data, and the standard deviation was used to describe the dispersion of the data. Qualitative variables were described by frequency distribution, and percentage frequency was used. The Kolmogorov-Smirnov test was used to check the normal distribution of data. An independent t-test and Chi-square test were used to compare the two groups in terms of different variables. Data analysis was carried out using SPSS version 22.

## 4. Results

**Figure 1.**
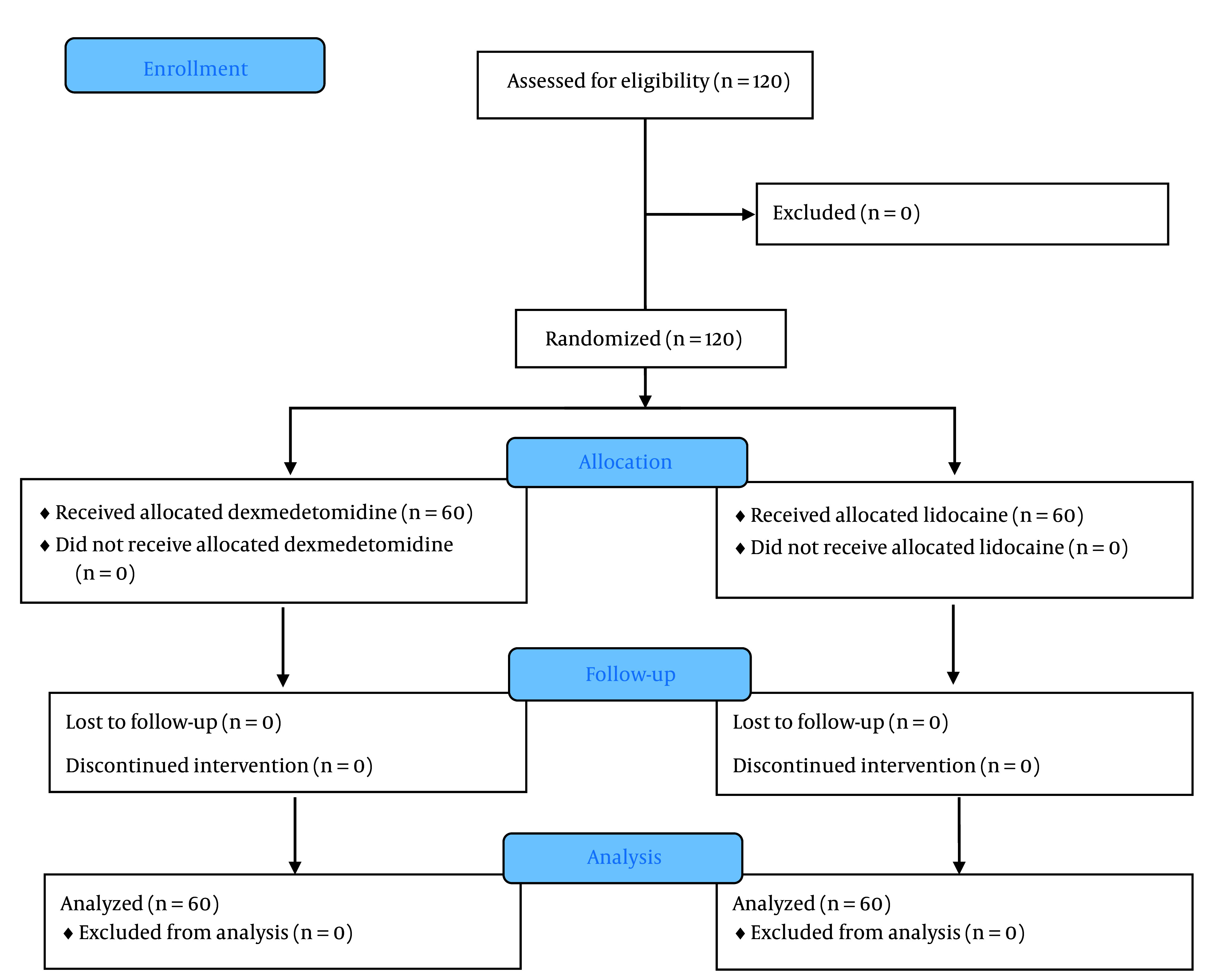
Consort diagram

In this study, 120 patients were examined for entry into the study and then divided into two groups of 60. During the intervention and follow-up, no one was excluded (Figure 1). 

Table 1 shows the patients' demographic characteristics in the lidocaine and dexmedetomidine groups.

**Table 1.** Comparison of Two Groups (Lidocaine and Dexmedetomidine) According to the Demographic Characteristics

Groups	Lidocaine	Dexmedetomidine	Significance Level
**Demographic characteristics**			
Mean age (y)	57.53 ± 11.34	56.90 ± 10.95	0.234
Weight (kg)	73.16 ± 14.4	68.81 ± 11.12	0.067
Male (%)	38 (63.3)	37 (61.7)	0.850
Female (%)	22 (36.7)	23 (38.3)	0.850
Time of procedure (min)	16.15 ± 1.39	16.18 ± 1.98	0.452

Based on the results, there were no significant differences between the two groups in gender, age, weight, and time of the procedure. Therefore, as far as the gender, age, and weight of the patients were concerned, the dexmedetomidine and the lidocaine groups were homogeneous.

**Figure 2.**
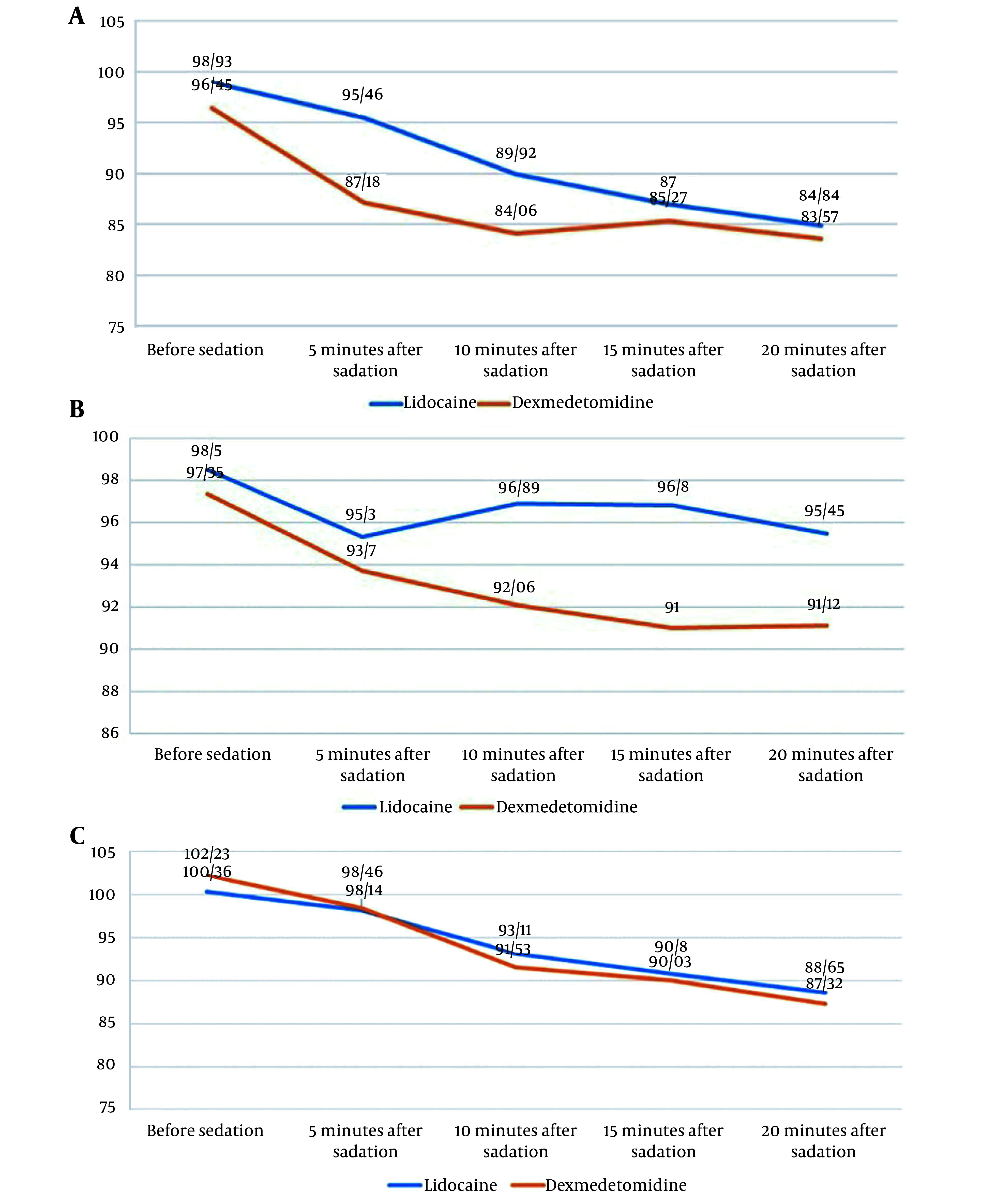
A, A Comparison of the two groups according to heart rate; B, comparison of the two groups according to SpO_2_; C, comparison of the two groups according to mean arterial blood pressure

Based on the results demonstrated in Figure 2A, the dexmedetomidine and the lidocaine groups were not significantly different regarding HR before sedation (P > 0.05). However, after sedation (from 5 to 20 minutes), these groups were significantly different in this regard (P < 0.05), and HR was lower in the dexmedetomidine group, but there was no case of bradycardia.

As can be seen in Figure 2B, with respect to the degree of arterial blood oxygen saturation before sedation and 5 minutes after sedation, the dexmedetomidine and the lidocaine groups were not significantly different (P > 0.05). However, we observed significant differences between them with respect to the degree of arterial blood oxygen saturation after sedation (from 10 to 20 minutes) (P < 0.05), and patients receiving dexmedetomidine had lower values.

According to Figure 2C, the dexmedetomidine and the lidocaine groups were not significantly different regarding the mean arterial blood pressure prior to sedation (P > 0.05). After sedation (from 5 to 20 minutes), the mean arterial blood pressure in both groups was not significantly different (P > 0.05).

Table 2 shows the degree of sedation among patients receiving lidocaine and dexmedetomidine before sedation and 5, 10, 15, and 20 minutes after sedation.

**Table 2.** Comparison of Two Groups (Lidocaine and Dexmedetomidine) in Terms of the Degree of Sedation

Groups		Lidocaine	Dexmedetomidine	Significance Level
**Degree of sedation**				
**Before sedation**	Score	1.86	1.92	0.435
**5 minutes after sedation**	Score	3.23	3.32	0.678
	Significance level	0.651	0.883	
**10 minutes after sedation**	Score	4.76	4.83	0.567
	Significance level	0.998	0.198	
**15 minutes after sedation**	Score	4.48	4.52	0.112
	Significance level	0.078	0.234	
**20 minutes after sedation**	Score	5.05	4.98	0.309
	Significance level	0.455	0.670	

The dexmedetomidine and the lidocaine groups were not significantly different regarding the degree of sedation (from 5 to 20 minutes) (P > 0.05).

Table 3 lists the pain score in the lidocaine and dexmedetomidine groups.

**Table 3.** Comparison of Two Groups (Lidocaine and Dexmedetomidine) According to Degree of Pain

Groups			Lidocaine	Dexmedetomidine	Total	Significance Level
**Degree of sedation**						
**Start of the procedure**	No pain	Distribution	17	17	34	0.325
		Percentage	28.33	28.33	28.33	
	Slight	Distribution	35	39	74	
		Percentage	58.33	65	61.67	
	Moderate	Distribution	8	4	12	
		Percentage	13.33	6.67	10	
	Severe	Distribution	0	0	0	
		Percentage	0	0	0	
**End of the procedure**	No pain	Distribution	19	22	41	0.034
		Percentage	31.66	63.67	32.5	
	Slight	Distribution	10	31	41	
		Percentage	16.68	51.66	25	
	Moderate	Distribution	31	7	38	
		Percentage	51.66	11.67	42.5	
	Severe	Distribution	0	0	0	
		Percentage	0	0	0	
	Significance level		0.265	0.001		
**One hour after the end of the** **procedure **	No pain	Distribution	20	22	42	0.362
		Percentage	33.33	36.67	35	
	Slight	Distribution	17	22	39	
		Percentage	28.34	36.66	32.5	
	Moderate	Distribution	23	16	39	
		Percentage	38.33	26.67	32.5	
	Severe	Distribution	0	0	0	
		Percentage	0	0	0	
	Significance level		0.02	0.041		

At the beginning of the procedure and one hour after the end of the procedure, the dexmedetomidine and the lidocaine groups were not significantly different in terms of the degree of pain (P > 0.05). At the end of the procedure, however, we observed a significant difference between them with regard to the degree of pain (P < 0.05), which was lower in the dexmedetomidine group.

Table 4 shows the patients' recovery time in the lidocaine and dexmedetomidine groups.

**Table 4.** Comparison of Two Groups (Lidocaine and Dexmedetomidine) According to Recovery Time

Groups		Lidocaine	Dexmedetomidine	Total	Significance Level
**Recovery time**					
**Fast **	Distribution	41	38	79	0.740
	Percentage	68.33	63.33	65.83	
**Medium**	Distribution	19	22	41	
	Percentage	31.67	36.67	34.17	
**Slow**	Distribution	0	0	0	
	Percentage	0	0	0	

The dexmedetomidine and the lidocaine groups were not significantly different regarding the recovery time (P > 0.05).

Table 5 shows the patients' nausea in the lidocaine and dexmedetomidine groups.

**Table 5.** Comparison of Two Groups (Lidocaine and Dexmedetomidine) According to Nausea

Groups		Lidocaine	Dexmedetomidine	Total	Significance Level
**Nausea**					
**No nausea**	Distribution	32	29	61	0.362
	Percentage	53.33	48.33	50.83	
**Mild**	Distribution	24	28	52	
	Percentage	40	46.66	43.34	
**Moderate**	Distribution	4	3	7	
	Percentage	6.67	5	5.83	
**Severe**	Distribution	0	0	0	
	Percentage	0	0	0	

Based on the results, 59 people (49.16%) had nausea. There was no significant difference between nausea in the two groups (P > 0.05). None of the patients had vomiting.

## 5. Discussion

The present study compared the effect of dexmedetomidine versus intravenous lidocaine on colonoscopy candidates under sedation with propofol-fentanyl. Based on the results obtained, heart rate was significantly lower in patients receiving dexmedetomidine. The groups receiving dexmedetomidine and lidocaine did not differ significantly in mean arterial blood pressure. The degree of pain at the end of the procedure was lower in patients receiving dexmedetomidine. Although the degree of sedation was higher in the dexmedetomidine group, the study groups were not significantly different in this regard.

In 2021 Xu et al. compared co-administration of lidocaine and dexmedetomidine with lidocaine or dexmedetomidine alone for reducing pain in patients undergoing laparoscopic hysterectomy. Their study included 160 patients following laparoscopic hysterectomy who were randomized into the following groups: C group (control group) receiving normal saline, L group receiving lidocaine, D group receiving dexmedetomidine, and LD group receiving lidocaine with dexmedetomidine. Compared with the C group, patients in the D and LD groups received lower VAS scores (P < 0.05). Lidocaine plus dexmedetomidine administration resulted in a significant reduction in postoperative pain, low PONV in laparoscopic hysterectomy patients, and improved postoperative patient sedation (21). These results corroborate our findings, according to which patients receiving dexmedetomidine obtained a lower VAS score and a higher degree of sedation.

In 2021, Ibrahim conducted a prospective randomized controlled trial (RCT) and compared colonoscopy sedation using lidocaine and dexmedetomidine as opposed to propofol. The groups in their study included the following: P (propofol) group: Patients were sedated by 50 - 100 mg propofol, and sedation was maintained with intravenous propofol injection of 25 - 75µ/kg/min; DL (dexmedetomidine-lidocaine) group: Patients were initially given a dose of dexmedetomidine 1 µ/kg for 10 minutes, followed by the injection of dexmedetomidine 0.2 - 0.7 µ/kg/h and lidocaine 1 mg/kg, followed by the injection of lidocaine 1.5 mg/kg/h. Both patient groups reported satisfaction with treatment; however, the group that received propofol reported greater satisfaction. The midazolam and fentanyl doses needed by patients in the DL group to achieve an appropriate sedation score were significantly higher. In addition, their heart rate was significantly lower, and their postoperative pain scores were significantly higher compared with the P group (18). In the present study, lidocaine and dexmedetomidine were administered to two separate groups, and it was observed that HR and SPO_2_ were lower in the dexmedetomidine group.

 In a 2020 prospective, double-blind RCT, Li et al. investigated using IV lidocaine for obese patients subjected to painless colonoscopy. In their study, 90 obese patients who had been scheduled for painless colonoscopy randomly received either lidocaine (group L) (1.5 mL grams/kg, then 2 mg/kg/hour, intravenous) or an identical amount of normal saline 0.9% (N group). Intraoperative sedation was provided by propofol. Patients in the L group experienced decreased oxygen desaturation episodes (1.49 ± 1.12) compared to the N group (2.11 ± 1.32). Patients in the L group needed less propofol, were awake for a shorter period, and stayed shorter in recovery (22). In the present study, the lidocaine group was compared with dexmedetomidine, and the lidocaine group experienced higher SPO_2_.

One of the major limitations of our study was related to the small sample size and its implementation in a single center. Therefore, future trials should use a larger sample size and a multi-center research design. Future research is also advised to use newer pharmaceutical compounds and compare them with existing routine methods in order to obtain more reliable pharmaceutical compounds for this procedure.

### 5.1. Conclusions

Although administration of both lidocaine and dexmedetomidine is associated with minimal hemodynamic changes, dexmedetomidine can provide more suitable and favorable conditions during and after colonoscopy by inducing a higher degree of sedation and more analgesia.

## Clinical Trial Registration Code:

IRCT20220706055402N2.
